# Control of Genome Integrity by RFC Complexes; Conductors of PCNA Loading onto and Unloading from Chromatin during DNA Replication

**DOI:** 10.3390/genes8020052

**Published:** 2017-01-26

**Authors:** Yasushi Shiomi, Hideo Nishitani

**Affiliations:** Graduate School of Life Science, University of Hyogo, Kamigori, Ako-gun, Hyogo 678-1297, Japan

**Keywords:** DNA replication, genome integrity, chromatin, PCNA, RFC complex, PCNA loader, PCNA unloader, RFC1, Ctf18, Elg1

## Abstract

During cell division, genome integrity is maintained by faithful DNA replication during S phase, followed by accurate segregation in mitosis. Many DNA metabolic events linked with DNA replication are also regulated throughout the cell cycle. In eukaryotes, the DNA sliding clamp, proliferating cell nuclear antigen (PCNA), acts on chromatin as a processivity factor for DNA polymerases. Since its discovery, many other PCNA binding partners have been identified that function during DNA replication, repair, recombination, chromatin remodeling, cohesion, and proteolysis in cell-cycle progression. PCNA not only recruits the proteins involved in such events, but it also actively controls their function as chromatin assembles. Therefore, control of PCNA-loading onto chromatin is fundamental for various replication-coupled reactions. PCNA is loaded onto chromatin by PCNA-loading replication factor C (RFC) complexes. Both RFC1-RFC and Ctf18-RFC fundamentally function as PCNA loaders. On the other hand, after DNA synthesis, PCNA must be removed from chromatin by Elg1-RFC. Functional defects in RFC complexes lead to chromosomal abnormalities. In this review, we summarize the structural and functional relationships among RFC complexes, and describe how the regulation of PCNA loading/unloading by RFC complexes contributes to maintaining genome integrity.

## 1. Introduction

Genome integrity requires precise chromosome duplication. Duplication of genomic DNA occurs only once during S phase in the eukaryotic cell cycle [1]. Before replication is initiated, replication origins are licensed for replication by minichromosome maintenance (MCM) 2–7 complex loading onto origin recognition complex (ORC)-bound origins, assisted by Cdc6 and Cdt1 [2]. The next step is activation of the origins and the formation of replication forks. The active DNA helicase, CMG complex (comprising Cdc45, MCM2-7, and Sld5(go), Psf1(ichi), Psf2(ni), and Psf3(san) (GINS) complex), unwinds double-stranded DNA, and DNA polymerases are recruited for replication [3]. Accompanying these events are several other important processes, including repair; recombination; chromatin formation, modification, and remodeling; as well as the maintenance of epigenetic information and the prevention of re-replication during replication fork progression. Moreover, sister chromatid cohesion must occur, as their alignment is required for faithful chromosome segregation [4,5].

To carry out these various activities, the DNA replication fork requires many proteins that form a large complex, the replisome, to facilitate the efficient initiation and elongation of DNA synthesis and chromatin-associated events [6]. Among these proteins, in eukaryotes, the DNA sliding clamp proliferating cell nuclear antigen (PCNA) plays a fundamental role in coordinating multiple events on the DNA [7]. To perform all of its functions, both loading the PCNA onto DNA and removing it from DNA must be precisely regulated. To achieve this, PCNA uses the molecular PCNA ring-opening machinery, replication factor C (RFC) complex [8].

Here, we first describe PCNA and then focus on how PCNA loading and unloading are regulated while coupled to DNA replication. In particular, we highlight the role of the RFC complex as a PCNA loader or unloader that conducts replication-linked processes, and discuss how these functions are orchestrated to maintain genome integrity.

## 2. PCNA, the DNA Sliding Clamp in Eukaryotic Cells

### 2.1. Structure and Primary Function of PCNA

DNA replicative polymerases, particularly polymerase δ/ε, require additional factors to support DNA replication [9]. The DNA sliding clamp, PCNA, tethers DNA polymerases, strengthens the interactions of the polymerases with the template DNA, and enhances their processivity up to 1000-fold [10,11,12], which makes PCNA an essential processivity factor for DNA replication. Many PCNA-binding factors that are involved in replication-coupled processes have been identified [7].

**Figure 1:**
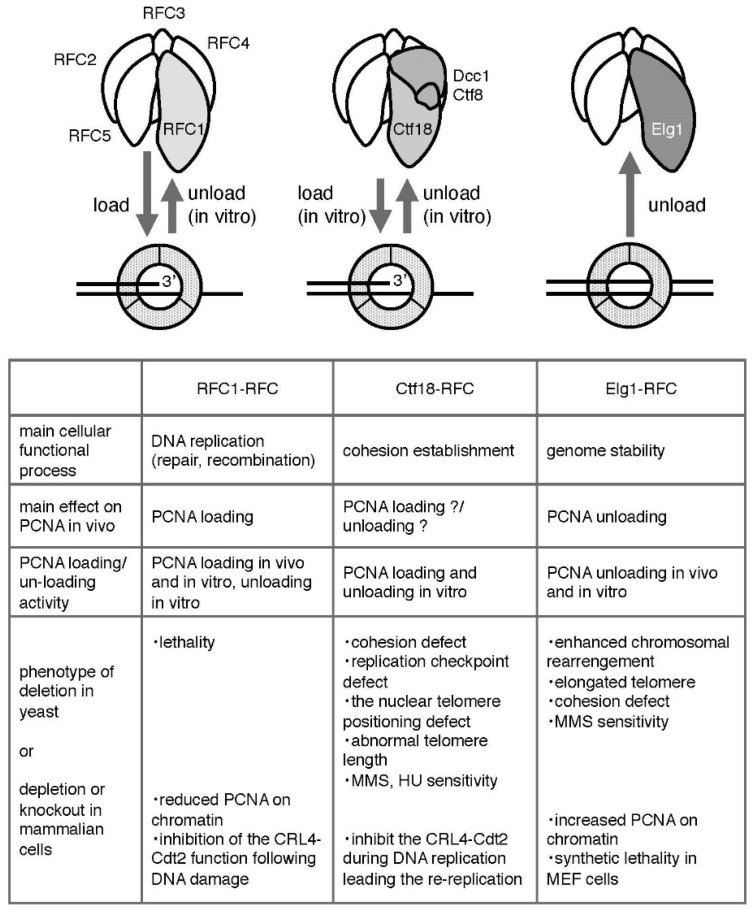
Summary of the functions of the three RFC complexes transacting on PCNA [17]. See text for details. The “?” marks in the table mean that the main effect of Ctf18-RFC (loading and unloading) on PCNA in vivo is not well understood.

**Figure 2:**
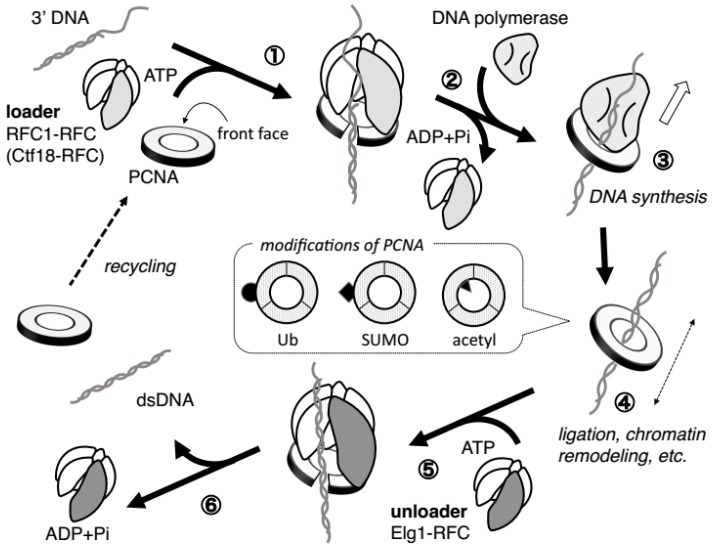
PCNA loading on and unloading from chromatin by RFC complexes during DNA synthesis. ① PCNA loader RFC1-RFC or Ctf18-RFC bind to PCNA and recognize the 3′ DNA template, and ATP binding triggers a conformational change of the RFC complex that allows for a tight interaction with PCNA and ring opening. ② ATP hydrolysis by the RFC loader complex is coupled with ring closure and the release of PCNA, finally encircling the DNA duplex. ③ DNA polymerases bind to chromatin-loaded PCNA, and DNA synthesis begins. ④ After the DNA synthesis is complete and DNA polymerase is released, PCNA recruits various enzymes for additional functions such as chromatin remodeling. PCNA slides along the double-stranded DNA to its functional sites. In ③ and ④, PCNA might be modified by mono-ubiquitin, poly-ubiquitin, SUMO (small ubiquitin-like modifier), or acetyl depending on the circumstances as illustrated in a dotted-line square (note that modification on single subunit of PCNA trimer is shown). ⑤ After the role of PCNA is completed, Elg1-RFC unloads PCNA from the double-stranded DNA in an ATP-dependent manner as a reverse reaction of PCNA loading. ⑥ During PCNA unloading, its modification might be removed so that it can be recycled. In this figure, nucleosomes and chromatin structures are omitted.

PCNA is a ring-shaped homo trimer, in which the three subunits assemble in a head-to-tail manner [13,14] (Figure 1). PCNA is loaded onto the DNA in an orientation-dependent manner. The association between PCNA and DNA is stable as PCNA encircles DNA and can slide freely along the DNA due to the polarity repelling effects between the inner surface of the PCNA ring and the DNA. The front face of PCNA has amino acid polarity that interacts with DNA polymerases and numerous other DNA metabolic enzymes, most of which have a PCNA-interacting protein (PIP)-motif, to recruit and tether them correctly to the DNA [15] (Figure 2). Because the regions that interact with these enzymes often overlap, PCNA switches its binding partner depending on the circumstances of the replication fork progression [7]. In addition, PCNA couples the initiation of DNA replication to ubiquitin-mediated proteolysis [16]. Thus, as a platform, PCNA plays an important role in the replisome by accommodating multiple processes at the replication fork [6].

### 2.2. Post-Translational Modifications of PCNA

Various PCNA modifications also regulate the replisome depending on specific circumstances during DNA replication [18] (Figure 2). Following DNA damage, PCNA is monoubiquitinated at K164 in a Rad18-Rad6-dependent manner, which switches the affinity of PCNA from replicative polymerases to damage-tolerant translesion synthesis polymerases, such as polymerase η [19,20]. The translesion synthesis (TLS) polymerases can bypass DNA damage to continue replication, though this method of damage bypass is prone to error [21,22]. In contrast, polyubiquitination of the same site by Mms2-Ubc13 and Rad5 leads to the repair through template switching, which is essentially an error-free mechanism [19]. PCNA can also be SUMOylated (small ubiquitin-like modifier) at the same site as K164 and K127 in a Ubc9- and Siz1-dependent manner [18]. SUMOylation mediates the repression of unwanted homologous recombination through recruitment of the helicase Srs2, which is well characterized in yeast [23,24]. SUMOylated PCNA also exists in vertebrates. Acetylation of PCNA appears to have a role in enhancing the processivity of associated polymerases, and promotes the removal of chromatin-bound PCNA and its degradation during nucleotide excision repair [25,26]. A recent study revealed that K20 at the inner surface of the PCNA ring is acetylated by cohesion acetyltransferase Eco1 in response to DNA damage, induces alteration of the PCNA structure, and stimulates homologous recombination [27].

### 2.3. PCNA Requires Ring-Opening Factors to Regulate Its ON–OFF DNA Binding

Because PCNA performs many aspects of DNA replication-associated events when loaded onto the DNA, PCNA loading onto the DNA must be strictly regulated. Conversely, when PCNA completes its role, it must leave (or unload from) the DNA to suppress illegitimate enzymatic reactions. To bind and leave the DNA, PCNA must temporarily open its closed ring, which is achieved by PCNA ring-opening machinery, RFC complexes [8].

## 3. Fundamental Features of RFC Complexes as PCNA Loaders/Unloaders

Eukaryotic cells have three RFC complexes that act on PCNA: RFC1-RFC, Ctf18-RFC, and Elg1-RFC, which essentially form hetero-pentameric complexes by sharing four small RFC subunits (RFC 2, 3, 4, and 5 [RFC2-5]), and each is distinguished by its largest subunit (i.e., RFC1, Ctf18, and Elg1, also called ATAD5 in human cells) [8] (Figure 1). Ctf18-RFC and Elg1-RFC are also called Ctf18-RFC-like complex (Ctf18-RLC) and Elg1-RFC-like complex (Elg1-RLC), respectively. All of these subunits, both large and small, belong to the AAA+ ATPase family [28,29]. The molecular morphologic similarity of these RFC complexes suggests that they all interact with PCNA and mediate the interactions between PCNA and DNA.

In addition to these complexes, eukaryotic cells have another RFC complex, Rad17-RFC (Rad24-RFC in *S. cerevisiae*), that acts to load the PCNA-like hetero-trimeric 9-1-1 complex (Rad9-Hus1-Rad1 in humans and Ddc1-Mec3-Rad17 in *S. cerevisiae*) at damaged DNA sites depending on checkpoint activation. We do not discuss this complex in this review and readers are referred to these excellent reviews [30,31].

### 3.1. RFC1-RFC

#### 3.1.1. Fundamental Features and Structure

**Figure 3:**
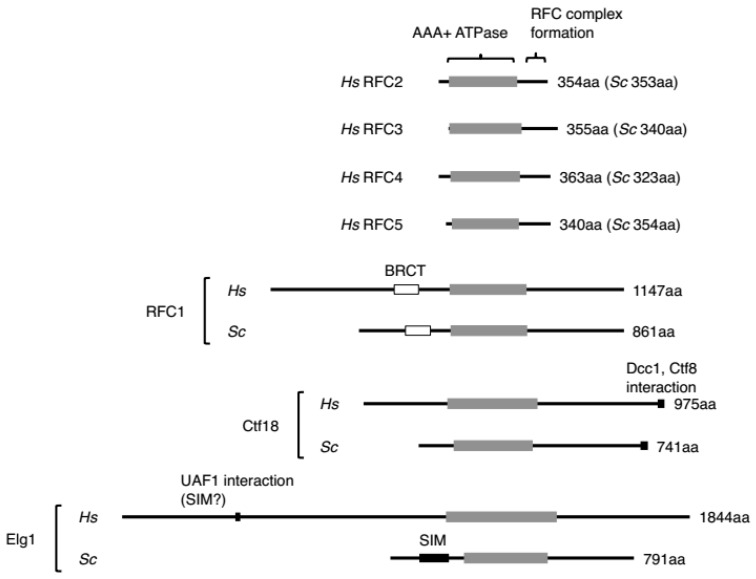
RFC subunit structures. The center of these subunits includes RFC boxes containing a P-loop, which is a general Walker-type ATPase motif. The C-terminal regions following the grey boxes representing the four small subunits (RFC2–5) are required for RFC complex formation. As for the largest RFC subunits, the domains required for complex formation are not well defined. The N-terminal of RFC1 contains a BRCT motif and the C-terminal of Ctf18 contains an interaction motif with Dcc1 and Ctf8. The N-terminal of Elg1 includes a SUMO (in *Sc*, called SIM) or UAF1 (in *Hs*) binding motif, which is potentially involved in PCNA binding. The UAF1 binding motif in *Hs* Elg1 likely has a role as a SIM, thus referred as “SIM?”. *Hs*: Homo sapiens, *Sc*: Saccharomyces cerevisiae.

A classic RFC complex, RFC1-RFC, comprises five subunits; the largest subunit is RFC1 and the four small subunits are RFC2-5. Common sequence motifs in these subunits are termed the RFC box, including the P-loop, a general Walker-type ATPase motif [32] (Figure 3). A yeast genetic study indicated that RFC1 is the only essential gene among the three RFC large subunits [33,34]. None of the other alternative RFC complexes is essential, alone or in combination [34]. RFC1 contains both N- and C-terminal extensions from the RFC box [35] (Figure 3). The C-terminals of the four small subunits and RFC1 are required to form the RFC1-RFC complex [36,37].

The biochemical activity of RFC1-RFC has been well analyzed, as described later, and structural analysis has provided details of the PCNA loading mechanism [38,39]. The crystal structure and electron microscopy images of the complex show that the five RFC1-RFC subunits are aligned in a circular shape with a gap between RFC1 and RFC5, making it well suited to interact with the PCNA ring [38,40,41] (Figure 1 and Figure 2). Therefore, RFC1-RFC is generally regarded as the standard for studying other RFC complexes.

#### 3.1.2. PCNA Loading/Unloading Activity of RFC1-RFC

The molecular role of RFC1-RFC for PCNA loading was first identified using purified complex from human HEK293 cells based on its requirement in the SV40 replication system in vitro [42,43]. Many biochemical analyses revealed that RFC1-RFC has multiple functions that allow for PCNA loading onto DNA at the 3′ primer/template junction in an ATP-dependent manner (Figure 2): PCNA binding, 3′ end of the primer DNA binding, and ATP binding trigger a conformational change of RFC1-RFC that allows it to bind tightly with PCNA and induce ring opening, and then ATP hydrolysis is associated with ring closure and release of the PCNA, which now encircles the DNA duplex. The binding partner of PCNA then switches from RFC1-RFC to DNA polymerase, and RFC1-RFC leaves the new DNA synthesizing complex, DNA polymerase/PCNA/DNA complex [6]. RFC1-RFC binds to a specific side of PCNA, the front face, and loads it in an orientation-dependent manner, so that the front face of PCNA that binds to its partner is oriented toward the elongating DNA (Figure 2). The ATP-driven PCNA-loading process by RFC1-RFC is discussed in detail in these excellent reviews [29,39,44,45,46].

In vitro experiments revealed that RFC1-RFC unloads PCNA from nicked or gapped circular DNA in an ATP-dependent manner as a reverse reaction of PCNA loading [47,48]. These observations led us to speculate that RFC1-RFC drives both PCNA loading and unloading during DNA replication. Whether unloading of PCNA occurs by RFC1-RFC in vivo, however, remains unclear. Interestingly, the subassembly complex of the four small subunits of RFC can also open PCNA and remove it from DNA, suggesting that all RFC complexes have the potential to unload PCNA [49].

### 3.2. Ctf18-RFC

#### 3.2.1. Fundamental Features and Structure

Ctf18-RFC was the second PCNA-conducting RFC complex identified. It is a hetero-pentamer formed by the large subunit Ctf18 and two additional subunits, Dcc1 and Ctf8, binding with the small subunits RFC2-5 [50,51,52,53] (Figure 1). These additional subunits are unique to this RFC, interact with the C-terminal end of Ctf18, and are conceivably located outside of the circle created by the other five subunits [54,55] (Figure 1). Ctf18, Dcc1, and Ctf8 are conserved from yeast to humans [53,56,57].

Early genetic analysis in *S. cerevisiae* revealed mutations in Ctf18 in screens for genes important for preventing chromosome loss, and it was thus termed chromosome transmission fidelity (previously called Chl12; chromosome loss) [58,59,60]. The two additional subunits, Dcc1 (defect of chromosome cohesion) and Ctf8, were also identified as genes required for chromosome segregation [59]. The absence of Ctf18, Ctf8, or Dcc1 singly or in combination leads to precocious sister chromatid separation accompanied by pre-anaphase accumulation of cells that depends on the spindle assembly checkpoint [50,61]. Ctf18 is located at replication forks with Ctf4 and Eco1, which are required to establish cohesion, coupled with PCNA recruitment [62]. These findings indicate that Ctf18-RFC is primarily required for sister chromatid cohesion, and might be involved in the regulation of PCNA on chromatin.

#### 3.2.2. PCNA Loading/Unloading Activity of Ctf18-RFC

Electron microscopy images of a recombinant pentameric Ctf18-RFC complex devoid of Dcc1 and Ctf8 subunits and designated here as Ctf18-RFC(5) are indistinguishable from RFC1-RFC, whose five subunits are aligned in a circle with a gap [54]. In addition, in the course of identifying human PCNA-interacting proteins by mass-spectrometric analysis, Ctf18 was identified together with RFC1 and four small subunits [63]. The results suggested that Ctf18-RFC also interacts with PCNA and functions as a PCNA loader. Actually, Ctf18-RFC loads PCNA onto nicked circular DNA or primed single-stranded DNA, which have a 3′ end, in vitro with both yeast and human recombinant proteins [52]. Consistent with this in vitro result, Ctf18 yeast mutants exhibit a reduced amount of chromatin-bound PCNA [62].

Interestingly, Ctf18-RFC(5) also effectively binds to and loads PCNA onto DNA, indicating that the two additional subunits are dispensable for PCNA-loading activity [54]. Thus, the Ctf18-RFC(5) supports DNA polymerase δ activity with PCNA on primed M13 single-stranded DNA in vitro, similar to human RFC1-RFC, demonstrating that Ctf18-RFC-loaded PCNA is functional. This Ctf18-RFC(5), however, cannot substitute for RFC1-RFC in the in vitro SV40 DNA replication system with a crude cell extract that includes the proteins required for replication, such as PCNA, RPA, and DNA polymerases, except RFC(s) [54]. Furthermore, in addition to loading activity, in vitro experiments with purified Ctf18-RFC showed unloading activity toward the primed DNA template [56]. The detailed activity of Ctf18-RFC for PCNA loading/unloading in the cells, however, is not fully understood.

### 3.3. Elg1-RFC

#### 3.3.1. Fundamental Features and Structure

Elg1-RFC is the most recently identified RFC complex forming a hetero-pentamer by RFC2-5 and the large subunit Elg1 [64,65,66] (Figure 1). Elg1 has a much longer N terminus compared with the other large subunits, especially in humans (Figure 3). Elg1 was first identified in a series of genetic screens in yeast, in which mutants exhibited various defects leading to genomic instability (hence, its name—enhanced levels of genome instability) [64,65,66,67,68,69,70,71,72]. In mammals, the corresponding gene was isolated as ATAD5 (ATPase Family, AAA Domain Containing 5).

#### 3.3.2. PCNA Unloading Activity of Elg1-RFC

**Figure 4:**
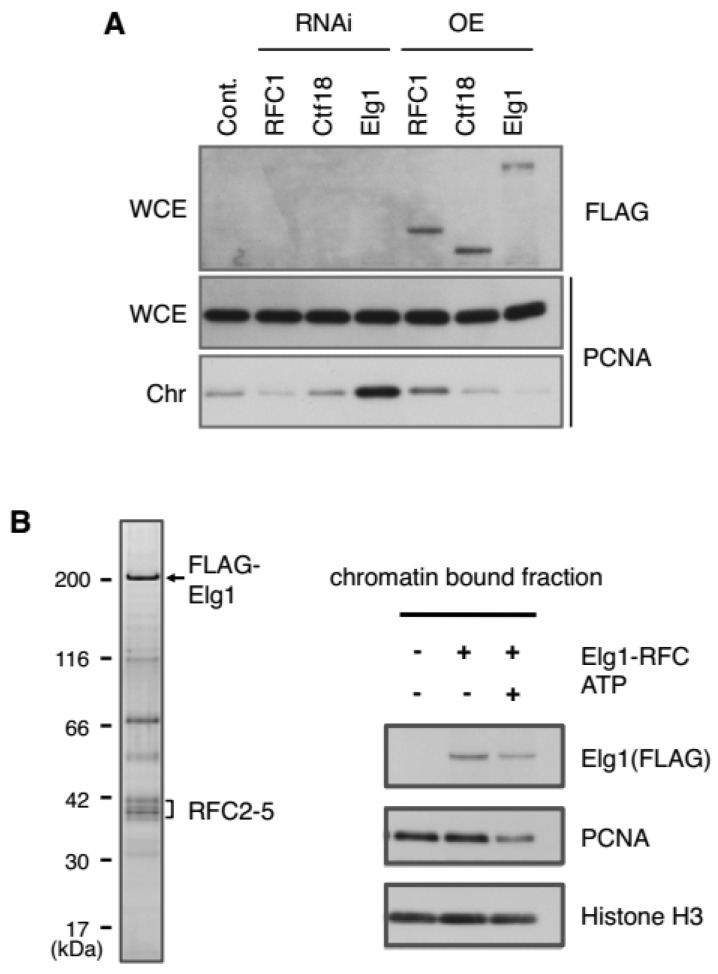
PCNA loading or unloading function of human RFC complexes. (**A**) Depletion by RNA interference (RNAi) or overexpression (OE) of the largest subunits of RFCs in human HEK293 cells. Whole cell extract (WCE) and chromatin-containing fractions (Chr) were prepared after centrifugation. The results demonstrated that RFC1-RFC and Elg1-RFC have a primary role in PCNA loading and unloading, respectively, in vivo. Depletion or overexpression of Ctf18 does not change the level of PCNA on chromatin in human cells; (**B**) PCNA unloading assay. Left panel: partially purified Elg1-RFC complex. HEK293T cells were co-transfected with FLAG-tagged Elg1 and RFC2-5, and complexes were purified with anti-FLAG antibody. Right panel: PCNA unloading assay. The purified Elg1-RFC was incubated with permeabilized cell nuclei containing PCNA-loaded chromatin in the presence or absence of ATP. The purified Elg1-RFC complexes unload PCNA from chromatin in an ATP-dependent manner.

Three independent groups demonstrated that Elg1-RFC functions as the major PCNA unloader during DNA replication, in both yeast and mammalian cells [73,74,75]. Suppression of Elg1 expression leads to an extreme accumulation of chromatin-bound PCNA and the corresponding PCNA foci are larger and more intense, indicating an extended lifespan of PCNA in replication factories. In contrast, overexpression of Elg1 results in a reduction of PCNA on chromatin (Figure 4A). Elg1 depletion also leads to an increase in the number of cells in S phase, indicating that abnormal levels of PCNA on chromatin affect cell-cycle progression [73,75].

By quantitative proteomic analysis of a yeast elg1 deletion strain, Kubota et al. initially observed a substantial accumulation of PCNA on the chromatin among other proteins [76]. They then used the auxin inducible degradation (AID)-Elg1 construct for timely depletion or induction of Elg1 in synchronized cell cultures [74,77]. Application of this system confirmed that the accumulation of PCNA and its SUMOylated forms on chromatin occurred in the course of the first cycle of DNA replication, and that lack of Elg1 resulted in a slight delay in S phase progression without checkpoint activation, similar to mammalian cells [74]. In the absence of Elg1, PCNA is not retained at specific sites on the chromatin, indicating that the Elg1-RFC unloads PCNA genome-wide, rather than only from specific chromosomal sites [78].

Partially purified Elg1-RFC from yeast or human cells unloads PCNA from chromatin isolated from an elg1 mutant in a yeast strain or from permeabilized nuclei from human cells in an ATP-dependent manner in vitro [74] (Figure 4B). These results strongly support that Elg1-RFC is a primary PCNA unloader. It remains unclear, however, whether Elg1-RFC is the only PCNA unloader during normal DNA replication, as Elg1 is not essential for cell division [34].

## 4. The Three RFC Complexes Contribute to Genomic Integrity by Controlling PCNA Loading/Unloading

### 4.1. Roles during DNA Replication Progression

As described above, the three RFC complexes likely share the roles of PCNA loading and/or unloading in vivo. RFC1-RFC, and probably Ctf18-RFC, primarily function as PCNA loaders and Elg1-RFC primarily functions as an unloader. Indeed, as shown Figure 4A, our results clearly demonstrated that depletion or overexpression of RFC1 or Elg1 in human cells have opposite effects on the PCNA levels on chromatin. In a knockdown experiment, depletion of RFC1 led to decreased PCNA levels on chromatin, while depletion of Elg1 led to increased PCNA levels. An overexpression experiment produced completely opposite results.

Once DNA replication is initiated, PCNA must be loaded onto DNA, both on leading and lagging strands. As expected, PCNA is detected almost twice as often on the lagging strand than on the leading strand at the replication fork [79]. On chromatin, PCNA plays multiple roles; first, it clamps the polymerase for DNA synthesis, and then it recruits many of the enzymes required for the following chromosomal events. The fact that RFC1, but not Ctf18 or Elg1, is essential also reflects the importance of PCNA loading by RFC1-RFC and its potential unloading activity [34]. While it appears that Ctf18-RFC can load PCNA on chromatin, it cannot substitute for RFC1 deletion, probably because the PCNA-loading activity of Ctf18-RFC is weaker than that of RFC1-RFC in vitro, or Ctf18-RFC may load PCNA for specific purposes such as for establishing cohesion [54,62].

Elg1-RFC is the PCNA unloader during normal DNA replication. The absence of Elg1 leads to various types of chromosome instability, such as DNA damage sensitivity, replication defects, enhanced homologous recombination, gross chromosomal rearrangements, chromosome maintenance defects, elongated telomeres, and cohesion defects [64,65,66,67,68,69,70,71,72]. In mammals, defects in corresponding ATAD5 likewise cause genomic instability and predisposition to cancer in human and mouse cells [80]. Mouse embryonic fibroblasts derived from ATAD5 heterozygous mice are highly sensitive to DNA damaging agents, demonstrating high levels of aneuploidy and genomic instability in response to DNA damage [81]. In addition, altered levels of recruitment of the PCNA-interacting proteins on chromatin were observed [75] (see Section 5). All these abnormalities may be due to enhanced retention of PCNA on chromatin [82].

PCNA unloading must be coupled with the completion of the chromosome replication process, because not only delayed but also precocious unloading would cause abnormalities in DNA replication and its associated chromosomal events. Actually, defects of the Okazaki fragment ligase Cdc9 in yeast leads to PCNA accumulation on chromatin, similar to the accumulation caused by a lack of Elg1 [78]. Thus, PCNA unloading is at least dependent upon completion of the Okazaki fragment ligation during DNA replication in S phase. The unloading of PCNA may be also dependent on the completion of the nucleosome assembly, because the absence of its assembly due to inhibition of histone supply causes PCNA to accumulate on chromatin [83].

Is Elg1-RFC the only PCNA unloader? Even in the absence of Elg1, the PCNA retained on the chromatin is eventually removed in the M phase [75]; therefore, the PCNA unloading function could conceivably be performed by RFC1-RFC and/or Ctf18-RFC, as suggested by their biochemical and genetic analyses. One possible regulation mechanism is modification of the largest subunits of RFCs, which may switch on either the PCNA loading or unloading activity. It is also possible that PCNA eventually spontaneously dissociates from DNA without the help of any unloaders [49]. Of course, there may be other novel pathways that can remove PCNA from the chromatin, such as acetylation-mediated removal and degradation of PCNA [26].

### 4.2. Roles in Sister Chromatid Cohesion

Ctf18-RFC is required for establishing sister chromatid cohesion, which may involve PCNA loading that aids the function of cohesion establishment factor Eco1. Eco1 associates with PCNA and promotes cohesion by acetylating the cohesion subunit Smc3 during S phase [84,85,86,87]. The fact that loss of either Ctf18, Dcc1, or Ctf8 causes cohesion defects and that these molecules form a DNA polymerase ε binding module suggest that sufficient levels of PCNA on the leading strand must be supplied by Ctf18-RFC, which would support polymerase ε and Eco1 acetylation activity [55,88,89].

A previous report indicated that Elg1 also plays a role in sister chromatid cohesion [71]. A yeast strain with deletion of Elg1, *elg1Δ*, exhibits precocious sister chromatid separation like the *ctf18Δ* mutant. Although the frequency is lower than that of the Ctf18-deleted strain, the *elg1Δ* strain is synthetic lethal with the cohesion mutants *scc1* or *smc1*. It is probable that inefficient PCNA unloading also affects cohesion establishment. Eco1 is recruited by PCNA, and PCNA SUMOylation appears to counteract Eco1 activity [90]. It is therefore possible that the excess PCNA SUMOylation observed in an *elg1Δ* mutant on chromatin interferes with the function of Eco1 in establishing cohesion. Indeed, a yeast Elg1 mutation that leads to over-SUMOylated PCNA, also causes a cohesion defect [90].

### 4.3. Roles for Proteolysis to Prevent DNA Re-Replication

Regulation of PCNA loading and unloading also has an important role in the once-per-cell-cycle replication. Chromatin-loaded PCNA activates the ubiquitin ligase CRL4-Cdt2 to prevent re-replication in the same cell cycle [16,91]. Cdt1 is a factor that is required for licensing of replication origins in G1 phase [92]. Cdt1 has a PIP-degron composed of a PIP-box sequence and downstream basic amino acid(s) [93,94]. When PCNA is loaded on chromatin upon the initiation of S phase, Cdt1 associates through its PIP-box, exposes the PIP-degron to CRL4-Cdt2, and is ubiquitinated for degradation. Cyclin-dependent kinase (CDK) inhibitor p21 and histone H4K20 mono methyltransferase Set8, which are also involved in the regulation of origin licensing, have PIP-degrons, and are also degraded by the same mechanism [95,96,97,98,99,100,101]. These proteins begin to re-accumulate around the end of S phase or G2 phase, when all of the PCNA is unloaded. Therefore, timely degradation and accumulation of these proteins are important for correct regulation of DNA replication, which is likely ensured by correct PCNA loading and unloading in the cell cycle. Ctf18-RFC is involved in CRL4-Cdt2 recruitment to the site of PCNA foci to degrade Cdt1, because RNA interference treatment of Ctf18, but not other large subunits, leads to defects in CRL4-Cdt2 recruitment at the replication fork. In contrast, RFC1-RFC contributes to CRL4-Cdt2 activation not during S phase, but following UV damage [102].

### 4.4. Roles of RFCs in Other Events

Several lines of experiments demonstrated that RFC complexes are involved in DNA repair processes. For example, when cells are irradiated with UV or treated with DNA-damaging reagents, the DNA repair reaction occurs and PCNA accumulates at the DNA-damaged sites, even though the cells are not in S phase. Studies of nucleotide excision repair, base excision repair, and mismatch repair have all demonstrated indispensable roles of RFC1-RFC in a DNA repair reaction to load PCNA and DNA pol δ/ε repair synthesis [103,104,105,106,107,108,109,110,111,112,113,114]. Elg1 is also involved in the DNA damage response [80,115]. It is not known, however, whether Elg1-RFC unloads PCNA after repair synthesis of the excised DNA damage site. A method termed enrichment and sequencing of protein-associated nascent DNA (eSPAN) was developed to discriminate proteins enriched at either the nascent leading or lagging strands. This method revealed that in yeast cells, PCNA is unloaded from the lagging strands upon stalling replication fork with hydroxyurea, and this process is dependent on Elg1 [79]. Cells deficient in PCNA unloading (*elg1Δ*) exhibit increased spontaneous chromosome breaks; hence, Elg1 contributes to genome stability when the supply of nucleotides is limited or when the replication fork encounters obstacles that cause replication stress.

Several studies revealed other aspects of Ctf18-RFC function that may be distinct from its function in sister chromatid cohesion. In a genome-wide specific screen for mutants affecting replication initiation, Ctf18 was newly identified and shown to physically interact with ORC, Cdt1, and MCM proteins. Furthermore, depletion of Ctf18 reduces pre-RC formation during the M-to-G1 phase transition, prevents S phase entry, and retards S phase progression [116]. Ctf18 is also essential for activating the DNA replication checkpoint upon the replication stress response [76,117,118]. Additionally, Dcc1 and Ctf8 are required for replication checkpoint activation, and for proper telomere length regulation and telomere intra-nuclear positioning [119]. Ctf18-RFC associates with DNA polymerase ε mediated by Dcc1 and Ctf8 at defective replication forks for activating the S phase checkpoint [88,89]. The Ctf18-RFC complex is also important for replication fork velocity and this effect seems to be linked to its major role in sister chromatid cohesion [120]. These findings represent new aspects of Ctf18-RFC′s roles. It is not yet fully elucidated, however, how these roles are related to its PCNA loading and unloading activity.

## 5. RFC Complexes May Play Roles beyond PCNA Loading/Unloading

### 5.1. Extended Region of the Large Subunits of RFCs

All large subunits of RFCs, especially human RFCs, have extensions in both the N-terminal and C-terminal regions from the RFC boxes (Figure 3). The extended regions of the large subunit likely have multiple roles, such as modulating PCNA loading/unloading, coupling the PCNA on chromatin with other events, as well as a role completely separate from its PCNA loading/unloading activities.

The N-terminal extension of RFC1 is not essential for cell viability, nor is it required for in vitro clamp loading activity, but removal of this region results in DNA damage sensitivity in vivo, suggesting that it has additional roles outside of its primary function as a PCNA loader [37,121]. The N-terminal extension of RFC1 contains a region that shares homology with DNA ligases, known as the BRCA1 C-terminal (BRCT) domain, though it does not have ligase activity. The structure of the human BRCT domain in solution suggested a binding model between BRCT and 5′-phosphorylated double-stranded DNA [122].

Ctf18 has an extended region in the C-terminus. As mentioned, the C-terminal end of Ctf18 interacts with Dcc1 and Ctf8 and forms a DNA polymerase ε binding module that is conserved from yeast to human cells and is important for activating the DNA replication checkpoint [88,89].

Although the interacting domain on Elg1 was not mapped, a recent finding showed that the Drosophila KAT6 Enok acetyltransferase complex interacts with Elg1 and inhibits its unloading activity [123]. The N terminal region of yeast Elg1 has a SUMO-interacting motif (SIM) at the N-terminal, and Elg1-RFC preferentially interacts and unloads SUMOylated PCNA from chromatin [73]. The N-terminal domain of yeast Elg1 might make a crucial contribution to PCNA unloading, because this domain interacts with PCNA and is important for the in vivo function of Elg1. Indeed, cells expressing Elg1 lacking the N-terminal 215 amino acids exhibit increased methyl methanesulfonate sensitivity compared with wild-type cells, but less methyl methanesulfonate (MMS) than an *elg1Δ* mutant [124]. The SIM at the N-terminus of Elg1 interacts with its target SUMOylated PCNA (and also unmodified PCNA), which helps Elg1-RFC bind PCNA strongly through all five subunits and open the PCNA ring to release it from chromatin. Because PCNA must be unloaded from double-stranded DNA passing through the PCNA ring, the unloading steps might not be a simple reverse reaction of PCNA loading [78].

Human Elg1 has an extremely extended N-terminal region, whose full amino acid length is 2.3 times longer than that of yeast Elg1 (*Hs* 1844 aa vs. *Sc* 791 aa; Figure 3). Human Elg1 also has a SIM in the N-terminal region. In contrast to yeast Elg1, however, the motif interacts with a SUMO-like domain in the deubiquitination factor UAF1 [125]. Thus, human Elg1 regulates PCNA deubiquitination by recruiting the USP1-UAF1 complex to ubiquitinated PCNA on chromatin. The different reactions of N-terminal Elg1 between yeast and human cells may reflect the difference in the levels of modification of PCNA between the species. In both cases, the N-terminal region may help to detect modified PCNA on chromatin and to facilitate its unloading. This process may be coupled with removal of the modification, as the loading of modified PCNA at a new site may bring about an irregular reaction by modified PCNA on chromatin.

### 5.2. RFC Complexes Interact with and Regulate Proteins Other than PCNA

The RFC complexes reported so far have various functions through their interactions with other factors. RFC1 interacts with DNA ligase I and negatively regulates its activity [126]. RFC1 binds directly to Asf1, a histone deposition protein, and histone deacetylase 1, and may play a role in replication-coupled chromatin remodeling or replication fork progression [127,128]. Additionally, RFC1 is suggested to regulate transcription, as it interacts with several transcription factors [129,130,131]. We demonstrated that RFC1-RFC and Ctf18-RFC interact with polymerase η, but RFC1-RFC inhibits its activity and Ctf18-RFC stimulates its activity in vitro [132]. Ctf18 also interacts with DNA polymerase ε to stimulate DNA synthesis activity [88,89]. RFC1-RFC and Ctf18-RFC appear to interact with the E3 ubiquitin ligase CRL4-Cdt2. Especially, Ctf18-RFC plays a role to recruit CRL4-Cdt2 to PCNA foci during DNA replication [102]. Thus, RFC complexes have more roles beyond PCNA loading/unloading and fulfill multiple functions.

Elg1 depletion in human cells leads to changes in the chromatin-bound levels of chromatin proteins. Elg1-depleted cells have decreased levels of proteins, such as RanGEF RCC1, SMC3, HBO1, SNF2H, HP1α, and Rif1 [75]. Most of these proteins bind to chromatin and correlate with chromatin remodeling behind the replication fork. In contrast, the chromatin levels of factors more directly involved in DNA replication, such as both PCNA-binding proteins, polymerase δ, DNA ligase I, MSH2, and non-PCNA binding protein Mcm6, remain the same after Elg1 depletion. These findings suggest that DNA replication processes such as Okazaki fragment maturation can be fulfilled correctly even in the absence of Elg1, but PCNA remains on the chromatin behind the active replisomes. Such unremoved PCNA could inhibit the association of chromatin formation, modification, or modeling factors. It is also possible that the extended N-terminus of Elg1 contains unidentified domains that interact with and recruit factors for chromatin transactions. Defects in Elg1 would induce changes in chromosomal stability. Analysis of cells in which endogenous Elg1 is replaced with nested deletion constructs will be required to define the role of the N-terminal domain.

## 6. Conclusions and Perspective

PCNA loading and unloading must repeatedly occur to initiate DNA synthesis and after the completion of every Okazaki fragment, as well as at replication fork termination. Three PCNA-conducting RFC complexes share the role of ensuring appropriate PCNA loading or unloading. In addition, all three RFC complexes have additional functions other than PCNA loading/unloading. It is unclear, however, why all eukaryotic cells require three similar RFC complexes. Given that RFC1-RFC and Elg1-RFC have primary roles in PCNA loading and unloading, respectively, it remains to be clarified how strictly the labor of PCNA loading/unloading is divided and shared by the three RFC complexes. Many questions remain regarding the detailed functions of the three RFC complexes to maintain genome integrity.
